# Transcriptional circuits of the glioma microenvironment: FOSL2 as a master regulator of the pathological niche

**DOI:** 10.1016/j.gendis.2026.102234

**Published:** 2026-05-12

**Authors:** Jia'ning Zhang, Chao Guan, Zechen Wang, Lizheng Jin, Zhen Lin, Jin Wen, Guangliang Chen, Pin Guo

**Affiliations:** a Department of Neurosurgery, The Affiliated Hospital of Qingdao University, Qingdao, Shandong 266000, China; b Department of Medical Oncology, Cancer Center, Zhejiang Provincial People's Hospital (Affiliated People's Hospital), Hangzhou Medical College, Hangzhou, Zhejiang 310014 China; c Department of Cardiovascular Medicine, Mayo Clinic, Rochester, MN 55905, USA; d Department of Medical Oncology, Fudan University Shanghai Cancer Center, Shanghai 200032, China; e Department of Oncology, Shanghai Medical College, Fudan University, Shanghai 200032, China

Glioblastoma (GBM) remains a lethal malignancy with median survival largely unchanged for decades. Beyond tumor cell-autonomous genetics, high-grade glioma is increasingly viewed as a breakdown of the neural niche, in which myeloid cells can comprise up to half of the tumor mass.^1^^,^^2^ However, myeloid-targeted approaches, including colony-stimulating factor 1 receptor (CSF1R) inhibition, have shown limited clinical benefit, highlighting a central challenge: glioma-associated macrophages (GAMs) are not a uniform population and cannot be reduced to an M1/M2 framework.^1^ Yu et al^1^ addressed this problem by combining single-cell RNA sequencing and single-cell assay for transposase-accessible chromatin sequencing to define a stable pathogenic myeloid state in glioma, termed malignancy-associated glioma macrophages (mGAMs). Defined by a Fos-like antigen 2 (FOSL2, also known as Fra-2)-centered regulon rather than surface markers alone, mGAMs orchestrate immunosuppressive and pro-invasive programs within the tumor microenvironment.^1^

The glioma niche is a dynamic ecosystem in which malignant cells interact continuously with immune cells, vascular networks, and the extracellular matrix (ECM) to promote tumor growth, invasion, and immunosuppression. Within this context, FOSL2 emerges as a master regulator that links angiogenesis, invasion, and immunosuppression to a single transcriptional node (Fig. 1). A member of the activator protein 1 (AP-1) family, FOSL2 appears to function as a stress-response hub activated by metabolic and physical pressures, particularly hypoxia.^1^ Together, studies by Yu et al, Wu et al, and Chen et al support a FOSL2-centered cross-compartment program, although they define related rather than identical myeloid states.^1^, ^2^, ^3^

**Figure 1:**
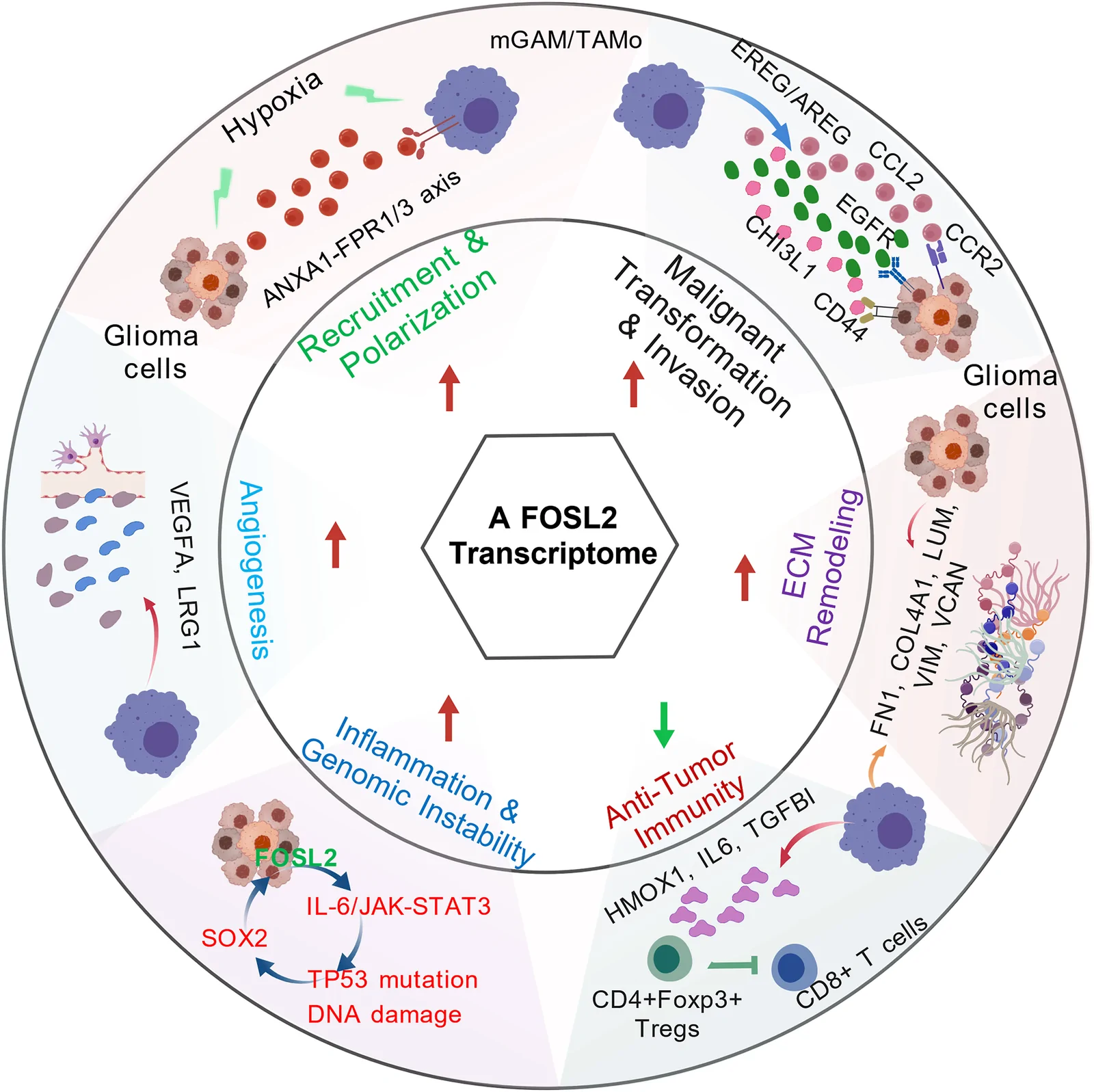
A panoramic circuit view of the FOSL2-orchestrated pathological niche in glioma. Glioma progression is driven by cross-cellular transcriptional programs organized around an FOSL2-centered regulon that integrates environmental stress, particularly hypoxia, with tumor-intrinsic genomic programs. From this regulatory hub emanate interconnected functional branches: i) Myeloid recruitment and polarization, whereby hypoxia-induced cues promote monocyte infiltration and differentiation into pathogenic macrophage states^2^^,^^3^; ii) Malignant transformation and invasion, in which niche-reprogrammed tumor-associated monocytes release EREG and AREG to activate EGFR signaling in glioma cells and reinforce mesenchymal and invasive programs^1^, ^2^, ^3^; iii) Suppression of anti-tumor immunity, driven by ANXA1–FPR1/3-dependent recruitment and polarization of immunosuppressive myeloid cells, with activation of HMOX1-, IL-6-, and TGFBI-associated programs linked to Treg expansion and impaired CD8^+^ T cell function^1^^,^^5^; iv) Extracellular matrix (ECM) remodeling, where a RUNX1–NPM1–FOSL2 transcriptional complex in tumor cells, together with secretory programs in mGAMs, induces deposition of matrix components, resulting in stromal stiffening^1^^,^^4^; v) Chronic inflammation and genomic instability, sustained by a SOX2–FOSL2–IL6 signaling axis associated with persistent inflammatory signaling and DNA-damage/genome-instability programs,^5^ although whether this branch operates in the same manner in FOSL2-high glioma niches remains to be established; and vi) Angiogenesis, supported by pro-angiogenic mediators, including VEGFA and LRG1, identified in a related monocyte-derived FOSL2-linked program.^1^ Together, these branches form a self-reinforcing pathological circuit that stabilizes the glioma niche and drives disease progression. The figure was created with BioGDP.com. ANXA1, annexin A1; AREG, amphiregulin; CCL2, C–C motif chemokine ligand 2; CCR2, C–C chemokine receptor type 2; CHI3L1, chitinase-3-like protein 1; ECM, extracellular matrix; EREG, epiregulin; EGFR, epidermal growth factor receptor; FOSL2, FOS-like antigen 2; FN1, fibronectin 1; FPR1/3, formyl peptide receptor 1/3; HMOX1, heme oxygenase 1; IL6, interleukin-6; JAK, Janus kinase; LRG1, leucine-rich alpha-2-glycoprotein 1; LUM, lumican; mGAMs, malignancy-associated glioma macrophages; NPM1, nucleophosmin 1; RUNX1, Runt-related transcription factor 1; SOX2, SRY-box transcription factor 2; STAT3, signal transducer and activator of transcription 3; TAMo, tumor-associated monocytes; TGFBI, transforming growth factor beta-induced; TP53, tumor protein p53; Tregs, regulatory T cells; VCAN, versican; VEGFA, vascular endothelial growth factor A; VIM, vimentin; COL4A1, collagen type IV alpha 1 chain.

Mechanistically, recent work suggests that this FOSL2 axis is progressively engaged during glioma progression, linking hypoxic stress to myeloid recruitment and pathogenic state formation. Wu et al show that hypoxia stabilizes hypoxia-inducible factor 1α (HIF-1α) in tumor cells, which in turn induces FOSL2 and promotes the secretion of annexin A1 (ANXA1).^2^ ANXA1 can engage formyl peptide receptor 1/3 (FPR1/3) receptors on bone marrow-derived monocytes/macrophages, facilitating recruitment and polarization.^2^ In parallel, a proposed “natural evolution signature” may drive progression of lower-grade gliomas toward a GBM-like state, a transition that appears to depend on recruitment of bone marrow-derived monocytes.^2^ Yu et al further show that recruited monocytes mature into an mGAM state characterized by high ANXA1 and heme oxygenase 1 (HMOX1) expression.^1^ Once established, mGAMs suppress anti-tumor immunity while promoting tumor invasion. Functional evidence indicates that mGAMs induce expansion of CD4^+^FOXP3^+^ regulatory T cells,^1^ thereby helping establish an immunosuppressive “cold” niche that may limit responses to checkpoint blockade. Beyond these immune effects, mGAMs also contribute to ECM remodeling by producing structural components, including vimentin and versican.^1^ Consistent with this finding, Cui et al show that ECM remodeling can stiffen the microenvironment, limiting T cell infiltration and providing a scaffold for tumor cell migration, while runt-related transcription factor 1 (RUNX1) complexes with nucleophosmin 1 (NPM1) in tumor cells maintain chromatin accessibility and enable FOSL2 to bind to and activate key ECM genes.^4^ Collectively, these studies support a model in which FOSL2 coordinates transcriptional programs in both tumor and myeloid compartments, thereby linking hypoxia-driven stress responses to immune suppression and matrix remodeling within the glioma niche (Fig. 1).

The FOSL2-centered regulon emerges as a central organizing node in a multicomponent pathological circuit that links environmental stress signals and tumor-intrinsic programs to glioma progression (Fig. 1). A key consequence of this circuit may be promotion of a mesenchymal-like tumor state associated with therapy resistance and poor prognosis. Chen et al^3^ identified tumor-associated monocytes, a related blood-derived myeloid program rather than a proven equivalent of mGAMs, that selectively express the epidermal growth factor receptor (EGFR) ligands epiregulin (EREG) and amphiregulin (AREG). These FOSL2-regulated ligands activate EGFR signaling in adjacent glioma cells and promote mesenchymal tumor programs.^3^ Chen et al also reported expression of the pro-angiogenic factors vascular endothelial growth factor A (VEGFA) and leucine-rich alpha-2-glycoprotein 1 (LRG1) in tumor-associated monocytes, supporting the angiogenesis branch shown in Figure 1, although whether these outputs are shared across all FOSL2-high myeloid states remains unresolved. Yu et al^1^ further show that mGAMs are enriched in hypoxic and necrotic niches of high-grade tumors, and that the mGAM transcriptional signature correlates with tumor grade rather than isocitrate dehydrogenase (IDH) mutation status, supporting mGAM accumulation as a core feature of glioma progression.^1^

In line with the “natural coevolution” model proposed by Wu et al,^2^ FOSL2-high tumor cells and infiltrating macrophages may form a feed-forward circuit in which hypoxia promotes monocyte recruitment and pathogenic myeloid differentiation, which in turn reinforces trophic signaling, invasion, necrosis, and further hypoxic stress. Beyond these microenvironmental effects, Njouendou et al^5^ linked the SRY-box transcription factor 2 (SOX2)–FOSL2–interleukin-6 (IL-6) axis to tumor-promoting inflammation and DNA-damage/genome-instability signatures. However, this circuit is best viewed as a working model rather than a fully resolved universal mechanism. Mechanistic support across these studies still relies in part on simplified systems, including long-term cultured cancer and monocyte cell lines, cytokine-polarized macrophage co-cultures, and orthotopic xenograft models in immunocompromised mice, which do not fully recapitulate the immune-competent and spatially heterogeneous glioma niche. In addition, causality between tumor evolution, myeloid remodeling, and mesenchymal transition remains incompletely resolved, and proposed bidirectional reinforcement between mesenchymal tumor cells and pathogenic myeloid states requires more direct testing. The ontogeny and clinical generalizability of these macrophage programs also remain incompletely defined, particularly given the possibility that distinct blood-derived and brain-resident myeloid populations may converge on similar pathogenic states under niche pressure. These considerations underscore the need for validation in larger, independent, and clinically diverse cohorts, including independent clinical validation of proposed surrogate markers such as ANXA1 and HMOX1.

Despite these limitations, the FOSL2-centered framework retains translational value by shifting attention from broad myeloid depletion to selective disruption of pathogenic programs. Marker-based approaches, including detection of mGAM-associated markers such as ANXA1 and HMOX1, may help identify tumors in which these circuits are engaged. The model highlights potential intervention points, particularly ANXA1–FPR signaling and ECM remodeling. Translation will nevertheless require careful prioritization, because FOSL2 itself may be difficult to target directly as a transcriptional hub, ANXA1-FPR signaling is biologically pleiotropic, and ECM-directed strategies are likely to be context-dependent. More broadly, the FOSL2-centered network may provide a useful framework for understanding how stress-responsive programs organize multicellular pathology in glioma and for identifying convergent therapeutic vulnerabilities.

## CRediT authorship contribution statement

**Jia'ning Zhang:** Writing – original draft. **Chao Guan:** Conceptualization. **Zechen Wang:** Data curation. **Lizheng Jin:** Software. **Zhen Lin:** Methodology. **Jin Wen:** Supervision. **Guangliang Chen:** Writing – review & editing, Visualization. **Pin Guo:** Visualization, Funding acquisition.

## Funding

This study was supported by the 10.13039/501100001809National Natural Science Foundation of China (No. 82573874, 81502151) and the 10.13039/501100007129Natural Science Foundation of Shandong Province, China (No. ZR2020MH261).

## Conflict of interests

The authors declared no conflict of interests.
